# Biosafety risk assessment for production of candidate vaccine viruses to protect humans from zoonotic highly pathogenic avian influenza viruses

**DOI:** 10.1111/irv.12698

**Published:** 2019-10-28

**Authors:** Li‐Mei Chen, Ruben O. Donis, David L. Suarez, David E. Wentworth, Richard Webby, Othmar G. Engelhardt, David E. Swayne

**Affiliations:** ^1^ Virology, Surveillance, and Diagnosis Branch Influenza Division National Center for Immunization and Respiratory Disease Centers for Disease Control and Prevention (CDC) Atlanta GA USA; ^2^ Exotic and Emerging Avian Viral Diseases Research Unit Agricultural Research Service U.S. National Poultry Research Center U.S. Department of Agriculture Athens GA USA; ^3^ Department of Infectious Diseases St Jude Children's Research Hospital Memphis TN USA; ^4^ Division of Virology National Institute for Biological Standards and Control Potters Bar UK; ^5^ Present address: IDT‐Biologika Rockville MD USA; ^6^ Present address: Biomedical Advanced Research and Development Authority Department of Health and Human Services Washington DC USA

**Keywords:** avian influenza, biosafety, candidate vaccine viruses, influenza vaccine, pandemics, pre‐pandemic

## Abstract

A major lesson learned from the public health response to the 2009 H1N1 pandemic was the need to shorten the vaccine delivery timeline to achieve the best pandemic mitigation results. A gap analysis of previous pre‐pandemic vaccine development activities identified possible changes in the Select Agent exclusion process that would maintain safety and shorten the timeline to develop candidate vaccine viruses (CVVs) for use in pandemic vaccine manufacture. Here, we review the biosafety characteristics of CVVs developed in the past 15 years to support a shortened preparedness timeline for A(H5) and A(H7) subtype highly pathogenic avian influenza (HPAI) CVVs. Extensive biosafety experimental evidence supported recent changes in the implementation of Select Agent regulations that eliminated the mandatory chicken pathotype testing requirements and expedited distribution of CVVs to shorten pre‐pandemic and pandemic vaccine manufacturing by up to 3 weeks.

## 1 INTRODUCTION

Highly pathogenic avian influenza (HPAI) viruses have the potential to cause zoonotic infections and to acquire human‐to‐human transmissibility, leading to a pandemic. Vaccination is the principal public health intervention to mitigate an emerging pandemic. Effective pandemic mitigation depends on achieving high vaccination coverage before the pandemic virus becomes widespread.^1^ The efficacy of licensed influenza vaccines depends on a high level of structural similarity between the hemagglutinins (HA) of vaccine and circulating viruses. Therefore, pandemic vaccines with structurally well‐matched HA antigens must be produced and administered as soon as possible after an emerging pandemic is detected. The National Pandemic Influenza Strategy calls for the United States (US) Department of Health and Human Services (DHHS) to maintain an updated library of CVVs and a strategic stockpile of vaccines to protect critical infrastructure in a pandemic emergency.^2^ The *Pandemic Influenza Plan 2017 Update* includes expectations for DHHS and its partners to maintain a high level of readiness to start immunizing the US population with a well‐matched pandemic vaccine within 4 months of a pandemic declaration.^3^ Achieving this challenging pandemic vaccination goal requires aggressive time management in all vaccine development and manufacturing steps, including rapid development of a pandemic CVV and its immediate distribution to vaccine manufacturers by the World Health Organization and its (international) partners.

## 2 PREPARATION AND DISTRIBUTION OF CVVS AGAINST HPAI TO MANUFACTURERS

Most of the influenza vaccine supply for the United States is produced by growing viruses in embryonated chicken eggs. Pre‐pandemic and pandemic vaccines for HPAI viruses developed using these technologies must be produced using attenuated CVV seeds that support worker safety during manufacturing.^4^ CVVs derived from HPAI viruses for pandemic influenza preparedness (PIP) are generated using reverse genetic technology to remove the multibasic amino acid motif from the cleavage site of the HA, which is the major determinant of high pathogenicity in chickens; that is, HPAI virus.^5^, ^6^, ^7^, ^8^ Attenuated CVVs (with a monobasic amino acid HA cleavage site) are engineered by reverse genetics and characterized at public health laboratories under quality system regulations in compliance with Food and Drug Administration (FDA) and World Health Organization (WHO) guidance and subsequently transferred to vaccine manufacturers for development of vaccine virus seeds per current good manufacturing practice (cGMP) standards.^9^, ^10^, ^11^, ^12^

**Figure 1:**
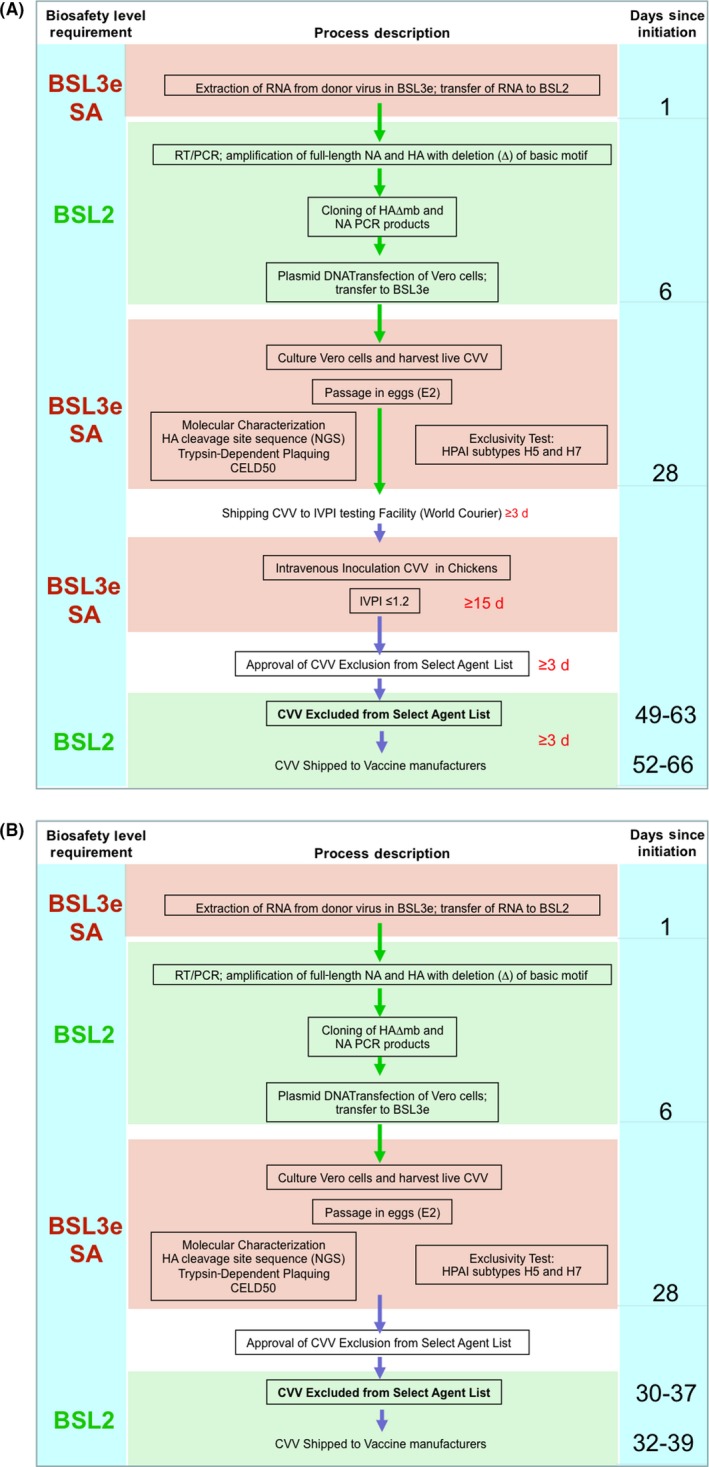
Development of CVVs against HPAI for pandemic preparedness. Schematic of major process steps and corresponding timeline under 2005 biosafety regulatory requirements in compliance with the Select Agent Program (A) and under the revised regulatory policy implemented in 2018 based on cumulative CVV safety data (B)

Currently, possession and transportation of wild‐type HPAI viruses in the United States are regulated under Select Agent rules (CFR 9 part 121) by the United States Department of Agriculture (USDA) Agricultural Select Agent Program.^13^ Furthermore, CVVs that are engineered with the attenuating monobasic HA cleavage site of an HPAI virus were considered Select Agents. However, CVVs with multibasic‐deleted HA can be used at a lower Biosafety Level after exclusion from the Select Agent list per CFR9 121.3e guidance.^14^ Exclusion from the Select Agent list was granted by USDA after review of the CVV information package with all the necessary experimental data supporting the loss of virulence for chickens and other phenotypic properties characteristic of low pathogenicity avian influenza (LPAI) viruses (Table 1, Figure 1A). This article describes the rationale and benefits of recent policy changes in the regulation of Select Agents in relation to development of CVVs for pandemic influenza preparedness and response purposes.^15^

**Table 1:** Biosafety risk assessment of pandemic CVV for exclusion from Select Agents list^14^, ^54^

Risk element	Parameter	Testing method	Outcome specification
Genomic composition	Source of all genes in construct; description of modification	Reference source material for viruses, plasmids, etc	Description of gene composition of recombinant/attenuated strain
HA activation by host proteases	Complete nucleotide sequence analysis of the entire HA gene and analysis of the amino acid motif at the HA cleavage site	Standard laboratory methods	Confirmation of expected sequence for attenuated strain. Demonstration of HA cleavage site that is consistent with LPAI virus
	Plaque characterization on chicken embryo fibroblast (CEF) cells (or other suitable cell lines) without trypsin	Test duplicate dilutions of strain in CEF or other appropriate cells with and without trypsin	Demonstration of inability to form clearly defined plaques in the absence of trypsin
	Plaque characterization on CEF cells (or other suitable cell lines) with trypsin	Determine plaque‐forming units/ml of representative product	Demonstration of ability to form viral plaques in the presence of trypsin
Lethality in poultry	Pathogenicity testing in chickens^a^	As described in the current OIE Manual of Standards for Diagnostic Tests and Vaccines	Confirmation of LPAI phenotype in chickens

a Risk assessment parameter is not required if the in vitro testing data meet the requirements of the new guidance.^54^

## 3 DHHS PANDEMIC VACCINE RESPONSE PLAN

A major lesson learned from the public health response to the 2009 H1N1 influenza pandemic resulted from the unexpected early start (August 2009) of the second wave of virus circulation and disease occurring approximately 6‐8 weeks before vaccination started (October 5, 2009), thereby weakening the impact of vaccination on reduction of disease burden.^16^ Shortening the timeline for development of CVVs for HPAI viruses would improve the timeliness of future pandemic vaccine deployments in response to an emerging HPAI that is easily transmitted among humans. To this end, the US Select Agent Program received requests to expedite pandemic vaccine development and production by improving operational plans and policies.^15^ The US government requirements established in 2005 for the process of exclusion of new CVVs derived from HPAI from the Agricultural Select Agent list have impacted the timeliness of distribution of CVVs to vaccine manufacturers.^17^ The resulting timelines for production of H5N1 vaccine affected the Strategic National Stockpile and could have delayed the public health response in a future pandemic.

Candidate vaccine viruses designed to protect from HPAI for pandemic preparedness have been produced by reverse genetic technology at three different laboratories in the United States (Food and Drug Administration [FDA], United States Center for Disease Control and Prevention [CDC], and National Institutes of Health/Saint Jude Children's Hospital [SJ]), one in the United Kingdom (National Institute for Biological Standards and Control [NIBSC]), one in Japan (National Institute of Infectious Diseases [NIID]), and one in China (Chinese National Influenza Center [CNIC], China Center for Disease Control and Prevention). Although the major virulence determinant for poultry (ie, multibasic cleavage site) is removed from the recombinant CVVs, current regulations require that viruses containing HA sequences from HPAI be created, propagated, and stored as Select Agents (SA) in BSL‐3 enhanced laboratory facilities, regardless of the structural or functional properties of their HA cleavage site. Compliance with the Select Agent regulations prior to the February 2018 revision required that CVVs be treated as SA (requiring additional specific approved forms and shipping requirements) until intravenous chicken pathogenicity testing showed attenuation and these data were submitted to USDA as part of SA exclusion process^18^ (Figure 1A). The pathogenicity results in chickens inoculated by the intravenous route, and other virus characterization data were included in the SA exclusion request submitted to the USDA Select Agent Program. With tight coordination and favorable circumstances, the animal studies performed after completion of CVV stock production in the laboratory added approximately 3‐5 weeks to the timeline to finalize the SA exclusion process (Figure 1A). If vaccine manufacturers could receive CVVs designed to protect against the emerging HPAI viruses immediately after SA exclusion based on the viruses' *in vitro* characterization is completed, the first doses of vaccine could be available for pandemic mitigation several weeks sooner. Therefore, expedited alternative approaches to assess the biosafety of CVVs derived from HPAI viruses were prioritized by the relevant federal government agencies.

## 4 BIOSAFETY RECORD OF CVVs SINCE 2004

The regulatory policy framework for conducting biosafety risk assessment supporting Agricultural Select Agent (ASA) exclusion of CVVs against HPAI was initially developed in 2003‐2004 and published in 2005.^13^ Although early studies showed that viruses engineered with a monobasic HA cleavage site equivalent to that of LPAI viruses were avirulent in chickens, it was not clear whether this approach would consistently yield viruses from diverse lineages with a similar safety profile,^5^, ^6^, ^19^, ^20^, ^21^, ^22^ particularly when applied to newly emerged HPAI viruses. Therefore, newly developed CVVs are regulated by the USDA as ASA per 9CFR 121^13^ and subsequently excluded from the ASA list following a prescribed regulatory pathway. Exclusion from the USDA ASA list per CFR9 121.3e was based on *in vitro* and *in vivo* characterization data. The Select Agent regulations implemented in 2005 required intravenous challenge study in chickens, which entailed intravenous (IV) inoculation of CVV stock into 10 chickens, 6 weeks of age, to determine morbidity and mortality per OIE protocol with intravenous pathogenicity index (IVPI) < 1.2, or intravenous inoculation into eight chickens, 4‐to‐8 weeks of age, with mortality less than 75% for exclusion from Select Agent rule.^18^, ^23^

In vitro characterization data inform three risk elements, as follows:

- Genome composition. All CVVs against HPAI tested so far were derived by plasmid‐based reverse genetics and contained six internal genes (PB2, PB1, PA, NP, M, and NS) derived from A/Puerto Rico/8/34 (H1N1) (PR8), a human influenza virus extensively passaged in eggs and mice.^8^
- HA cleavage site analysis. The HA genes from all CVVs were derived from reverse genetic plasmids engineered to have a monobasic cleavage site consistent with LPAI viruses.^5^, ^7^ The second or higher passage of the CVV recovered from transfected cells is sequenced to confirm the monobasic cleavage site in the HA.
- Trypsin‐dependent plaque formation. The second or higher laboratory passage of the CVV recovered from transfected cells is analyzed by plaque assay on primary chicken embryo fibroblast or other cells in the presence or absence of trypsin in the culture. Candidate vaccine viruses have invariably been dependent on trypsin for plaque development, whereas HPAI viruses are invariably trypsin‐independent.

Since 2003, a total of 40 H5 or H7 proposed CVV were produced using this approach at three laboratories in the United States, one in the UK, one in Japan, and one in China, under quality systems compliant with the Code of Federal Regulations (CFR), Volume 21, Part 58 (Good Laboratory Practices [GLP]) and WHO guidance (Table 2).^12^, ^24^, ^25^, ^26^, ^27^, ^28^ Intravenous chicken lethality values for the 40 H5 and H7 proposed CVVs for public health use plus an additional 18 candidate veterinary vaccine viruses tested were invariably 0%, indicating lack of virulence (Table 2) and resembling the outcomes of the least virulent LPAI virus inoculations.^18^, ^23^, ^29^ Similarly, three H9 CVVs lacked virulence in chicken pathotyping, which was also consistent with their LPAI parent H9 viruses (data not shown).

**Table 2:** Proposed candidate vaccine viruses against highly pathogenic avian influenza for pandemic preparedness (A) per WHO recommendations (2004‐2015) or veterinary use (B)

Virus strain designation	Clade	Institution		SA excl.		Chicken lethality (%)^a^
(A) H5N1 CVV						
A/Vietnam/1203/2004 (CDC‐RG)	1		CDC		Yes	0
A/Vietnam/1203/2004 (SJRG‐161052)	1		SJ		Yes	0
A/Vietnam/1194/2004 (NIBRG‐14)	1		NIBSC		Yes	0
A/Vietnam/HN30408/05 x PR8 (research grade)	1		CDC		No^b^	0
A/Cambodia/R0405050/2007 (NIBRG‐88)	1.1		NIBSC		Yes	0
A/Cambodia/X0810301/2013 (IDCDC‐RG34B)	1.1.2		CDC		Yes	0
A/duck/Hunan/795/2002 (SJRG‐166614)	2.1.1		SJ		Yes	0
A/Indonesia/5/2005 (CDC‐RG2)	2.1.3.2		CDC		Yes	0
A/Indonesia/NIHRD11771/2011 (NIIDRG‐9)	2.1.3.2a		NIID		NR	0
A/bar‐headed goose/Qinghai/1A/2005 (SJRG‐163222)	2.2		SJ		Yes	0
A/whooper swan/Mongolia/244/2005 (SJRG‐163243)	2.2		SJ		Yes	0
A/chicken/India/NIV33487/2006 (IBCDC‐RG7)	2.2		CDC/NIV		Yes	0
A/turkey/Turkey/1/2005 (NIBRG‐23)	2.2.1		NIBSC		Yes	0
A/Egypt/321/2007 (IDCDC‐RG11)	2.2.1		CDC		Yes	0
A/Egypt/N03072/2010 (IDCDC‐RG29)	2.2.1		CDC		Yes	0
A/Egypt/3300‐NAMRU3/2008 (IDCDC‐RG13)	2.2.1.1		CDC		Yes	0
A/Egypt/N04915/2014 (NIBRG‐306)	2.2.1.2		NIBSC		NR^c^	0
A/common magpie/Hong Kong/5052/2007 (SJRG‐166615)	2.3.2.1		SJ		Yes	0
A/duck/Bangladesh/19097/2013 (SJ007)	2.3.2.1a		SJ		Yes	0
A/Hubei/1/2010 (IDCDC‐RG30)	2.3.2.1a		CDC		Yes	0
A/Barn‐Swallow/HK/D10‐1161/2010 (SJ‐003)	2.3.2.1b		SJ		Yes	0
A/duck/Viet Nam/NCVD‐1584/2012 (NIBRG‐301)	2.3.2.1c		NIBSC		NR	0
A/Anhui/1/2005 (IBCDC‐RG6)	2.3.4		CDC		Yes	0
A/Japanese white‐eye/HK/1038/2006 (SJRG‐164281)	2.3.4		SJ		Yes	0
A/chicken/Hong Kong/AP156/2008 (SJ‐002)	2.3.4		SJ		Yes	0
A/chicken/Bangladesh/11rs1984‐30/2011 (IDCDC‐RG36)	2.3.4.2		CDC		Yes	0
A/Guizhou/1/2013 (IDCDC‐RG35)	2.3.4.2		CDC/CNIC		Yes	0
A/Sichuan/26221/2014 (H5N6) IDCDC‐RG42A	2.3.4.4		CDC/CNIC		Yes	0
A/gyrfalcon/WA/41088‐6/2014(H5N8) IDCDC‐RG43A	2.3.4.4		CDC		Yes	0
A/duck/Hyogo/1/2016 (H5N6) (NIID‐001)	2.3.4.4		NIID		NR	0
A/Hubei/29578/2016(H5N6) (CNIC‐HB29578)	2.3.4.4		CNIC		NR	0
A/Fujian‐Sanyuan/21099/2017 (CNIC‐21099)	2.3.4.4		CNIC		NR	0
A/chicken/Vietnam/NCVD15A59/2015 (H5N6)	2.3.4.4		CDC		Yes	0
A/goose/Guiyang/337/2006 (SJRG‐165396)	4		SJ		Yes	0
A/chicken/Viet Nam/NCVD‐016/2008 (IDCDC‐RG12)	7.1		CDC		Yes	0
A/chicken/Viet Nam/NCDV‐03/2008 (IDCDC‐RG25A)	7.1		CDC		Yes	0
(A) H7N3 CVV						
A/Canada/rv444/2004 (H7N3) SJRG‐161984‐B	American		SJ		Yes	0
(A) H7N9 CVV						
A/Guangdong/17SF003/2016( NIBRG‐375)	Eurasia		NIBSC		Yes	0
A/Guangdong/17SF003/2016( CBER‐RG7C)	Eurasia		CBER		Yes	0
A/Guangdong/17SF003/2016(CNIC‐GD003)	Eurasia		CNIC		NR	0
(A) Parental viruses						
A/Puerto Rico/8/1934 (H1N1, reverse genetics)	Human		CDC		NA^d^	0
A/Vietnam/1203/04 (H5N1) wt	1		CDC		NA	100
A/Cambodia/X0810301/2013 (H5N1) (wt)	1.1.2		CDC		NA	100
A/Indonesia/05/2005 (H5N1) wt	2.1.3.2		CDC		NA	100
A/chicken/Bangladesh/11rs1984‐30/2011 (H5N1) (wt)	2.3.4.2		CDC		NA	100
(B) H5 (Veterinary Use Only)						
A/turkey/Ireland/1983	Eurasian		SEPRL		Yes	0
A/duck/BacLieuVietnam/09/2007	1.1		SEPRL		Yes	0
A/chicken/Indonesia/07/2003	2.1.1		SEPRL		Yes	0
A/chicken/West Java/SMI‐HAMD/2006	2.1.1		SEPRL		Yes	0
A/chicken/Konawe Selatan/BBVW 204/2007 (PR8 NA)	2.1.3		SEPRL		Yes	0
A/chicken/Konawe Selatan/BBVW 204/2007 (A/Egret/HongKong/757.2/02 NA)	2.1.3		SEPRL		Yes	0
A/chicken/Konawe Selatan/BBVW 204/2007 (A/chicken/Indonesia/07/2003 NA)	2.1.3		SEPRL		Yes	0
A/chicken/Pekalongan/BBVW‐208/2007	2.1.3		SEPRL		Yes	0
A/chicken/West Java/PWT‐WIJ/2006 (PR8 NA)	2.1.3.2		SEPRL		Yes	0
A/chicken/West Java/PWT‐WIJ/2006 (A/chicken/Indonesia/07/2003 NA)	2.1.3.2		SEPRL		Yes	0
A/chicken/West Java/PWT‐WIJ/2006 (A/Goose/Hong Kong/437.4/99 NA)	2.1.3.2		SEPRL		Yes	0
A/chicken/Garut/BBVW‐223/2007 (PR8 NA)	2.1.3.2		SEPRL		Yes	0
A/chicken/Garut/BBVW 223/2007 (A/chicken/Indonesia/07/2003 NA)	2.1.3.2		SEPRL		Yes	0
A/chicken/West Java (NGR)/30/2007	2.1.3.2		SEPRL		Yes	0
A/whooper swan/Mongolia/244/2005	2.2		SEPRL		Yes	0
A/chicken/Egypt/9403 NAMRU3/2007	2.2.1		SEPRL		Yes	0
A/Muscovy duck/HanamVietnam/84/2007	2.3.4.3		SEPRL		Yes	0
A/gyrfalcon/Washington/41088‐6/2014	2.3.4.4		SEPRL		Yes	0

a % mortality in 8‐chicken intravenous pathotyping test or OIE protocol (10 chickens).

b CVV produced in research laboratory conditions (non‐GLP).

c N.R. = no requested exclusion submitted to US Select Agent program.

d N.A. = not applicable.

The numerous CVVs developed to protect against H5 and H7 HPAI virus subtypes, comprising diverse HA and NA genetic lineages (Eurasian and North American) and divergent genetic clades (Table 2),^28^, ^30^ were produced by reverse genetic‐engineered reassortment with a human virus adapted for optimal growth in eggs, usually A/Puerto Rico/8/34 (PR8) or its derivatives.^31^, ^32^ Despite extensive genetic diversity in the HA/NA and some variation among backbone virus genes, all CVVs and veterinary vaccine viruses shared the phenotypic character of avirulence in chickens (Table 2).^33^ Taken together, these studies strongly support the absolute necessity of a basic amino acid cluster or insertion of additional amino acids at the cleavage site to impart high virulence for chickens to reassortant viruses with PR8 genetic background.

## 5 BIOSAFETY RISK ASSESSMENT FOR POULTRY: THE EVIDENCE SINCE 2004

PIP CVVs could pose a significant threat to the health of avian species in the event of accidental or intentional release of viruses that might depart from expected characteristics by: (a) retaining intrinsic virulence for birds; (b) reverting to a virulent state; or (c) becoming contaminated with a wild‐type HPAI virus in the laboratory. The risks posed by each of these pathways merit detailed analysis of the mitigation options.

### 5.1 Mitigating residual virulence risk

The body of knowledge on the biological properties of PIP CVVs in chickens has increased by at least an order of magnitude since the original CVV regulatory framework was established in the United States more than a decade ago 13 (Table 3). The three risk mitigation requirements for CVV exclusion from the ASA list are (a) sequence analysis of the cleavage site; (b) plaque formation in the presence and absence of trypsin; and (c) intravenous lethality testing in chickens. As shown in Table 2, 40 H5 and H7 proposed CVVs and 18 H5 veterinary vaccine viruses evaluated under this framework have shown 100% concordance among the three parameters. These findings are consistent with the well‐established importance of the multibasic and/or elongated cleavage site of the HA (molecular marker) for plaque formation in the absence of trypsin (*in vitro* marker) and the high virulence in chickens (*in vivo* IVPI).^34^, ^35^, ^36^, ^37^, ^38^, ^39^, ^40^, ^41^ The abundance of data indicates that H5/H7 CVV with monobasic cleavage site and trypsin‐dependent plaque phenotype would have a negligible potential to cause severe disease in chickens.

**Table 3:** Biosafety risks and mitigation strategies for PIP CVV production and use

Risk	Mitigation
Intrinsic CVV properties	
HA cleavage virulence factor	Nucleotide sequence of HA cleavage site designed per WHO Guidance^43^, ^a^ Monobasic HA cleavage site verified by sequencing The absence of non‐conserved amino acid insertions at cleavage site (eg, H7 subtype)^b^
HA cleavage without trypsin	Trypsin‐dependent plaque assay
Internal genes from HPAI imparting virulence by unknown mechanism	Sequence‐confirmed internal gene segments using NGS^a^, ^b^
Virulence phenotype by unknown mechanism	Chicken embryo lethality (CEL) test: Embryonic death is reduced by 10‐fold relative to HPAI counterpart at 48 h after inoculation^a^
CVV processing and handling	
Inadvertent introduction of HPAI H5Nx or H7Nx	Exclusivity testing by rtRT‐PCR for HPAI H5 and H7 genes^a^
	Trypsin‐dependent plaque assay
	Lethality for chicken embryo absent^a^

a Characteristic or testing not required by USDA APHIS to support exclusion of CVV from Select Agent list

b Testing by next‐generation sequencing (NGS) has become standard practice for CVV characterization in 2016, adding sensitivity to the risk mitigation program (lower analytical limit of detection).

### 5.2 Mitigating the risk of reversion to high virulence

HPAI viruses arise from subtype H5 and H7 LPAI ancestors upon sustained circulation in chickens, turkeys, quail, ostriches, and other terrestrial bird species. The key molecular event is the acquisition of mutations encoding multiple basic amino acids or insertion of amino acids at the cleavage site of the HA. However, the mechanisms and fitness drivers of these mutations are not well understood. It has been hypothesized that RNA secondary structures flanking the cleavage site may favor polymerase stuttering leading to insertional mutagenesis and codon duplication.^42^ Per WHO guidance for PIP CVV development, codons for the monobasic cleavage site of the HA should feature silent nucleotide changes to achieve the lowest possible frequency of bases that could contribute to these events, reducing the potential for polymerase errors leading to re‐creation of a multibasic amino acid cleavage site.^43^ All CVV prepared by the WHO Global Influenza Surveillance and Response System (GISRS) laboratories applied these concepts to design HA cleavage sites with the lowest probability of spontaneous reversion to multibasic cleavage sites. Additionally, the monobasic cleavage site sequence of new CVVs was re‐examined after serial passage (10X) in eggs and confirmed the absence of reversion to mutations coding for multibasic cleavage site.

### 5.3 Mitigating risk of laboratory contamination with wild‐type HPAI

CVVs with HA and NA genes selected to protect from HPAI viruses are generated from a set of reverse genetic (RG) plasmids encoding the 8 viral genomic segments upon transcription by host cell polymerase I promoter and terminator elements.^44^ Inadvertent introduction of RG plasmids encoding wild‐type HA from HPAI viruses to plasmid stock to be used in the preparation of CVV would yield mixed viral populations with potential virulence for chickens and other avian species. To mitigate this risk, recent CVVs have been generated from HA‐sequenced plasmid DNA preparations that originate from a single bacterial colony prepared in compliance with quality system regulations, including Good Laboratory Practice standards established by FDA.^45^ These regulations include the use of dedicated facilities and equipment, restricted facility access, trained personnel, gowning and environmental control, raw material qualification program, single product handling, approved protocols, document controls and batch records, and complete decontamination/line clearance between each new CVV produced.^45^ CVVs are generated by DNA transfection of qualified cell cultures from a cGMP cell bank tested for adventitious agents.^26^, ^46^ All product‐contact and raw materials are pre‐qualified to be free of pathogenic infectious agents. It is worth noting that the use of synthetic DNA, which has been used increasingly in the generation of CVVs, results in the absence of wild‐type HPAI HA genes in the production facility. Therefore, the risk of introducing a HPAI virus into the CVV production environment is extremely low to nearly zero. That said, a set of tests are performed after each CVV is produced to identify signals inconsistent with those that define the CVV as similar to LPAI viruses (Table 3), often including complete genome coding sequence analysis using a next‐generation sequencing (NGS) approach (at least 100X coverage) with verification of the monobasic HA cleavage site and PR8 internal gene segments; trypsin‐dependent plaque formation in cell monolayers; chicken embryo lethality test; and often an additional exclusivity test for CVV quality control consisting of a real‐time reverse transcription‐polymerase chain reaction (rtRT‐PCR) assay for H5 and H7 HA genes from HPAI virus lineages that is performed by an independent QA laboratory.^47^ Of note, next‐generation sequencing technologies enable the identification of minor species of RNA present in the population, exclusivity testing uses the most sensitive methods currently available to identify other gene segments, and trypsin‐dependent plaque formation and embryo lethality are used because, if a wild‐type HPAI virus was present, it would replicate more efficiently than the CVV under these conditions, so that even very low‐level contamination would be identified with confidence.

### 5.4 Potential risks to poultry health attributable to CVV development and use: evidence of low environmental impact potential

HPAI viruses are important agricultural pathogens. A recent outbreak in the United States resulted in the culling of over 50.4 million poultry and economic losses exceeding $3.3 billion.^48^ While pandemic mitigation is a high public health priority, preventing HPAI outbreaks in poultry is equally important for animal health. Pandemic influenza preparedness CVVs are used for the production of inactivated human vaccines according to processes licensed by the US Food and Drug Administration.^49^ The inactivation methods used by licensed vaccine manufacturers have been rigorously validated to insure loss of residual viral infectivity.^49^ Although the final vaccine product is considered free of live CVV, vaccine manufacturing processes require virus propagation in eggs or cell cultures. Vaccine manufacturing plants in the United States are required to contain the virus within the facility and reduce the probability of live virus release from the manufacturing facilities. This is achieved by chemical disinfection of liquid waste and physical treatment of solid waste materials (generally by incineration) in compliance with local, national and international guidance.^50^ However, mechanical failure or human errors can breach containment methods—despite all efforts to the contrary. In the event of live CVV release to the environment, birds (poultry or wild) may come in contact with live CVVs. To better understand the potential risk of PIP CVV for poultry, we evaluated the replication and shedding of 17 CVVs in chickens following simulated respiratory tract exposure (Table A1).^51^ To this end, groups of 8 to 10 birds were inoculated intranasally (IN) and evaluated for 14 days. At 2 or 3 days post‐challenge, oropharyngeal and cloacal swabs were collected from all IN‐challenged animals, while two birds were euthanized and tissues collected for histopathology, immunohistochemistry (IHC), and virus isolation. At 14 days post‐challenge, birds were euthanized and blood samples were collected for serology. Antibody responses to influenza antigens were evaluated by agar gel immune diffusion or ELISA tests. Fourteen recombinant A(H5N1), one A(H5N6), and one A(H5N8) viruses with PR8 internal genes were analyzed in this fashion and compared to the PR8 donor of internal genes as well as several parental wild‐type A(H5N1) donors for HA and NA (Table A1). Few of the CVVs showed evidence of infection after IN challenge. With rare exceptions, infectious CVVs were not present in swab samples at 3 dpi, and antibodies were not detected. In a few exceptional cases, shedding of virus was detected, but less frequently and of lower titers than is typically seen with respiratory tract infections by LPAI viruses in chickens.^23^, ^52^ In agreement with these findings, the IV‐challenged birds showed no evidence of clinical disease. In contrast, wild‐type H5N1 or H7N3 replicated efficiently after IN challenge with profuse shedding, causing 100% mortality, typical microscopic lesions with abundant viral antigen detected with immunohistochemical (IHC) methods in tissues (data not shown). Although the HA and NA of the PIP CVVs originate from viruses with high fitness in birds, the six internal genes from A/Puerto Rico/8/34 are likely the viral components responsible for imparting poor replication/shedding in birds.^5^ These phenotypic differences are expected to result in rapid elimination of CVVs from exposed poultry because the reproductive number would be substantially lower than 1 (*R*_0_ < 1).^53^ These data indicate that PIP CVVs against HPAI with a PR8 backbone pose minimal risk to the health and well‐being of poultry and other birds.

## 6 REGULATORY POLICY REVIEW TO IMPROVE PANDEMIC PREPAREDNESS AND PUBLIC HEALTH RESPONSES

A large number of CVVs with HA sequences from HPAI viruses engineered with a monobasic cleavage site have been generated and characterized in the past 14 years. Invariably, they showed loss of replicative fitness and virulence in chickens. This body of information provided a compelling basis to re‐evaluate the contribution of *in vivo* studies in chickens to virulence assessment of future CVVs prepared according to equivalent methods and protocols. In addition, the array of risk mitigation practices for production and characterization of PIP CVVs in the WHO GISRS laboratories have indicated that health risks to birds posed by these viruses are equivalent to or less than those of LPAI viruses, which are not regulated as ASA, but rather as BSL2 animal pathogens under CFR9 122 by the USDA.^14^ The BSL2 biosafety guidance includes substantial barriers to CVV release into the environment to prevent potential exposure of susceptible birds. Practices, safety equipment (primary barriers and personal protective equipment), and facilities are designed to contain the release of infectious CVV. Although avian (H1‐H16) LPAI, including H7N9 LPAI of waves 1‐4, and swine (H1 and H3) influenza A viruses have zoonotic potential, they are not subject to Select Agent rules. However, the same scientific process to reduce virulence and transmissibility for agricultural animals by utilizing the PR8 backbone could be incorporated in public health and veterinary medical CVV risk assessment process to determine whether fewer enhancements are needed at BSL‐2 than would be required for wild‐type parent viruses, especially for parent viruses of foreign origin.

The recently updated policy governing PIP CVV development 15, 54 had major positive impact on the availability of GLP‐compliant laboratory space to conduct CVV development in response to emergencies. Candidate vaccine viruses development can now be completed and viruses shipped to vaccine manufacturers within approximately 5 weeks, saving nearly 3 weeks relative to the 2005—compliant development timeline, mitigating risks of poorly performing CVV—including low antigen yield, egg adaptive mutations, and other features that might affect the timeliness and effectiveness of the pandemic vaccination campaign (Figure 1B).

In conclusion, the 2018 revision of the requirements for exclusion of CVV under CFR9 121.3(e) Agricultural Select Agent regulations significantly expedite the production and distribution of CVVs to support timely pandemic influenza vaccine development and production for clinical trials, enable stockpiling, and consequently promote rapid vaccine administration during a pandemic response.

## CONFLICT OF INTEREST

The authors declare no conflict of interest in this review.

## DISCLAIMER

The findings and conclusions in this article are those of the authors and do not necessarily represent the views of the Centers for Disease Control and Prevention or United States Department of Agriculture.

## APPENDIX A

**Table A1:** Replication and shedding characteristics of H5 and H7 CVVs in chickens by intranasal inoculation^a^

Virus	IVPI	OP swab^b^	Cloacal swab^b^	Serology^c^
H5 CVV				
A/Vietnam/1203/2004 (CDC‐RG)	0	1/8	0/8	4/8
A/VN/HN30408/05 x PR8	0	0/8	0/8	0/8
A/Indonesia/5/2005 (CDC‐RG2)	0	0/10	0/10	0/8
A/chicken/India/NIV33487/2006 (IBCDC‐RG7)	0	1/2	2/10 (1.0)	0/8
A/Egypt/321/2007 (IDCDC‐RG11)	0	0/10	0/10	0/8
A/Egypt/N03072/2010 (IDCDC‐RG29)	0	0/10	0/10	0/8
A/Egypt/3300‐NAMRU3/2008 (IDCDC‐RG13)	0	0/10	0/10	0/8
A/Hubei/1/2010 (IDCDC‐RG30)	0	0/10	0/10	0/8
A/Anhui/1/2005 (IBCDC‐RG6)	0	0/10	0/10	0/8
A/chicken/Vietnam/NCVD‐016/2008 (IDCDC‐RG12)	0	0/10	0/10	0/8
A/chicken/Vietnam/NCVD‐03/2008 (IDCDC‐RG25A)	0	0/10	0/10	0/8
A/chicken/Bangladesh/11rs1984‐30/2011 (IDCDC‐RG36)	0	0/10	0/10	0/8
A/Guizhou/1/2013 (IDCDC‐RG35)	0	1/10	0/10	0/8
A/Cambodia/X0810301/2013 (IDCDC‐RG34B)	0	1/10	0/10	1/8
A/Sichuan/26221/2014 (H5N6) IDCDC‐RG42A	0	0/10	0/10	0/8
A/gyrfalcon/Washingont/41088‐6/2014(H5N8)‐IDCDC‐RG43A	0	0/10	0/10	0/8
A/VN/HN30408/05 x PR8 (non‐GLP)	0	0/10	0/10	0/8
H7 CVV				
A/mallard/Netherlands/12/2000 (H7N7) (IBCDC‐1) Conventional reassortant from LPAI ancestor of HPAI virus	0	0/8	0/8	0/8
WT parent				
A/Vietnam/1203/04	2.98	8/8	8/8	na^d^
A/Cambodia/X0810301/2013	3.0	10/10	10/10	na
A/Indonesia/05/2005	3.0	2/2	2/2	na
A/chicken/Bangladesh/11rs1984‐30/2011	3.0	10/10	10/10	na

a The indicated number of 4‐ to 8‐wk‐old birds was inoculated with 10^6 EID50 of CVV (egg passage 2 or higher) or wild‐type virus in 0.05 ml allantoic fluid given into the nares. At 2 or 3 d post‐inoculation, swabs were collected and analyzed by virus isolation in embryonated eggs. At 14 d post‐inoculation, birds were bled and serum was tested by AGID to detect antibodies to avian influenza.

b Number of birds yielding infectious virus from oropharyngeal or cloacal swab/total number inoculated

c Number of birds with detectable levels of serum antibody/total number of birds sampled

d na = not available. Chickens died before sampling date established in experimental protocol.
